# Functional annotation of hypothetical proteins from the *Exiguobacterium antarcticum* strain B7 reveals proteins involved in adaptation to extreme environments, including high arsenic resistance

**DOI:** 10.1371/journal.pone.0198965

**Published:** 2018-06-25

**Authors:** Wana Lailan Oliveira da Costa, Carlos Leonardo de Aragão Araújo, Larissa Maranhão Dias, Lino César de Sousa Pereira, Jorianne Thyeska Castro Alves, Fabrício Almeida Araújo, Edson Luiz Folador, Isabel Henriques, Artur Silva, Adriana Ribeiro Carneiro Folador

**Affiliations:** 1 Laboratory of Genomic and Bioinformatics, Center of Genomics and System Biology, Institute of Biological Science, Federal University of Para, Belém, Pará, Brazil; 2 Biotechnology Center, Federal University of Paraiba, João Pessoa, Paraíba, Brazil; 3 Biology Department & CESAM, University of Aveiro, Aveiro, Portugal; Boston University, UNITED STATES

## Abstract

*Exiguobacterium antarcticum* strain B7 is a psychrophilic Gram-positive bacterium that possesses enzymes that can be used for several biotechnological applications. However, many proteins from its genome are considered hypothetical proteins (HPs). These functionally unknown proteins may indicate important functions regarding the biological role of this bacterium, and the use of bioinformatics tools can assist in the biological understanding of this organism through functional annotation analysis. Thus, our study aimed to assign functions to proteins previously described as HPs, present in the genome of *E*. *antarcticum* B7. We used an extensive *in silico* workflow combining several bioinformatics tools for function annotation, sub-cellular localization and physicochemical characterization, three-dimensional structure determination, and protein-protein interactions. This genome contains 2772 genes, of which 765 CDS were annotated as HPs. The amino acid sequences of all HPs were submitted to our workflow and we successfully attributed function to 132 HPs. We identified 11 proteins that play important roles in the mechanisms of adaptation to adverse environments, such as flagellar biosynthesis, biofilm formation, carotenoids biosynthesis, and others. In addition, three predicted HPs are possibly related to arsenic tolerance. Through an *in vitro* assay, we verified that *E*. *antarcticum* B7 can grow at high concentrations of this metal. The approach used was important to precisely assign function to proteins from diverse classes and to infer relationships with proteins with functions already described in the literature. This approach aims to produce a better understanding of the mechanism by which this bacterium adapts to extreme environments and to the finding of targets with biotechnological interest.

## Introduction

*Exiguobacterium* are Gram-positive mobile bacteria that have psychrophilic and thermophilic adaptations according to the environment they live in. Isolates from this genus can be found in the most variable environments, from glacial ice to temperate soils, and have the capacity to survive in a range of extreme temperatures and in effluents contaminated with heavy metals such as arsenic and chrome [1]. Most of the species, such as *Exiguobacterium antarcticum*, are extremophile microorganisms that produce several enzymes that are stable at a broad range of temperatures, with numerous industrial applications such as for biosensors, environmental bioremediation and pharmaceutical applications [2–9].

These characteristics have triggered biotechnological interest in these bacteria and have aroused the interest of researchers in the past few years to investigate the different proteins involved in cold-adaptation. As an example, the recent identification and structural and biochemical characterization of a novel esterase, *Ea*EST, from *E*. *antarcticum* B7, was due to the great commercial potential of cold-adapted esterases for industrial applications [10]. Another study has revealed the different quaternary structure of GH1 β-glucosidase from the *E*. *antarcticum* B7 structural basis for cold adaptation [11]. Additionally, Baraúna and colleagues (2016) have investigated the role of the FapR regulator of *E*. *antarcticum* B7 as the main protein responsible for the regulation of fatty acid synthesis during cold adaptation [12].

*E*. *antarcticum* strain B7 was the second species of the genus to have its genome completely sequenced and published, allowing genomics, transcriptomics and proteomics studies to have better understanding of microbial adaptation mechanisms [9,13–15]. However, there are still challenges to improve the understanding of these mechanisms. Thus, bioinformatics approaches can play an important role in improving the understanding of biological processes, the gene repertoire and gene regulation, including protein-protein interactions (PPI) [16,17].

Functional annotation is crucial for determining the function of proteins during proteome analysis. Meanwhile, the function of a considerable number of coding sequences still cannot be predicted. For this reason, these molecules are labelled hypothetical proteins (HPs). Most of these proteins are believed to play an important role in the cell, and their annotation can lead to knowledge about new structures, functions and pathways. Proteins with unknown function can be assigned by homology-based gene annotation due to the correlation with known proteins [18–22].

Several recent bioinformatics tools, such as the Conserved Domain Architecture Retrieval Tool (CDART), the Simple Modular Architecture Research Tool (SMART), CATH, Pfam, SUPERFAMILY and SVMProt, have been developed to assign functions to HPs from many species [21,23–27]. These tools are associated with all the data available in many databases using domain, family and ontology information to support protein function characterizations. In addition, the study of PPI using software for protein interaction searches, such as the STRING database [28], is essential for understanding the role of a protein in a biological network [21,29]. These interactions play an important role in cellular processes, and by studying them, an understanding of HP function and inferences about biological functions for these non-elucidated proteins can be reached [30,31]. Furthermore, three-dimensional modeling is important to associate structural information with the function of unknown proteins, through homology searches at the Protein Data Bank (PDB) [32].

The utilization of *in silico* approaches to the functional prediction of HPs has been successfully used in several bacterial species, such as *Vibrio cholerae*, *Neisseria gonorrhoeae*, *Clostridium difficile*, and *Staphylococcus aureus* [22,33–35]. Due to the relevance of *E*. *antarcticum* B7, the purpose of this work was to assign function to the hypothetical proteins present on the genome of this species for the identification of new proteins that may contribute to an improved understanding of the adaptation of this bacterium to the extreme environment and for new biotechnological targets, adopting an integrated workflow, containing conventional annotation programs allied to PPI analysis, and three-dimensional protein modeling.

## Materials and methods

### Retrieval of genome data

In this work, we used the *Exiguobacterium antarcticum* B7 genome. This strain was isolated from biofilms in Ginger Lake, King George Island, Antarctica. Its genome has 2,815,863 bp and 2772 genes. It was retrieved from the National Center for Biotechnology Information database—NCBI (https://www.ncbi.nlm.nih.gov/genome/) under accession number CP003063.1 [9]. Subsequently, a total of 765 coding sequences (CDS) annotated as hypothetical proteins were extracted from this genome, using Artemis software [36].

### Functional annotation of hypothetical proteins

To unveil the function of the HPs using the programs and databases described in S1 Table, we first submitted these proteins to annotation using the GO FEAT tool 1.0 to a preliminary prediction [37], using an *e-value* 1e^-03^. GO FEAT is a new, free, online platform for functional annotation based on the homology search analysis on multiple databases, such as protein (Uniprot) [38], genome annotation, domain and family (InterPro and Pfam) [25,39] databases and, a cross reference using NCBI (https://www.ncbi.nlm.nih.gov/) and EMBL (http://www.ebi.ac.uk/ena/) databases.

**Fig 1:**
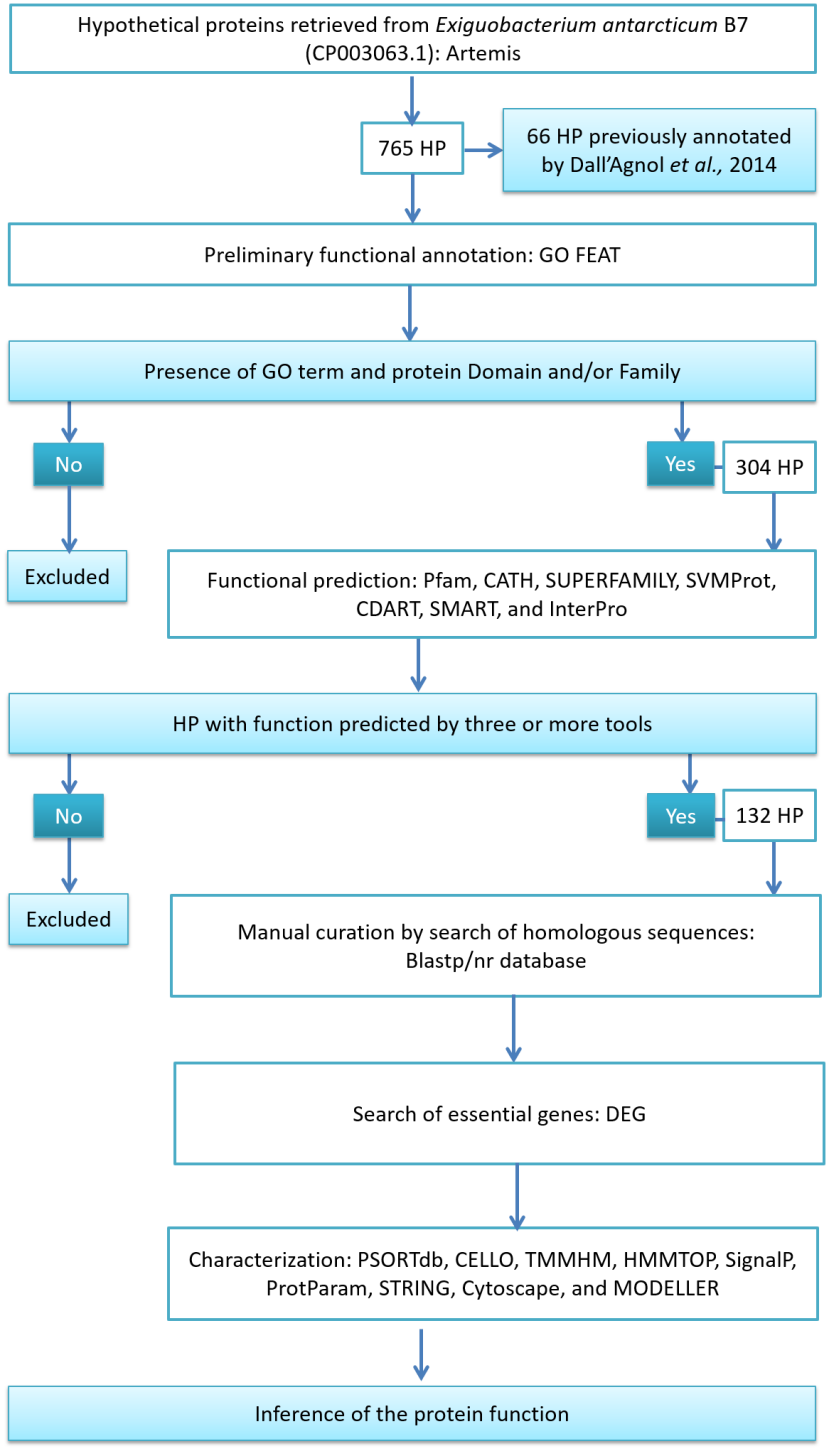
Workflow used for the annotation of the hypothetical proteins from *E*. *antarcticum* B7.

All HP-presenting products that described family and/or protein domains, according to the GO FEAT results, were then selected for further analysis using a variety of publicly available bioinformatics tools for domain and function assignment (Fig 1). CDART [24] and SMART 8.0 [26] were used to search conserved domains using the Conserved Domain Database (CDD), and protein function based on domain architecture, respectively. CATH 4.2 was used to classify the domains within structural hierarchy [40]. Pfam 31.0 [25], SUPERFAMILY 1.75 [23] and SVMProt [27] were used to classify the HPs into functional families to predict the function based on similarity. We also used InterPro 66.0 for motif detection, which uses the integration of numerous available databases for functional prediction. For all databases, we used default parameters.

Then, CDS were manually annotated by searching for homologous proteins from related organisms using the Basic Local Alignment Search Tool (BLAST) against the NCBI non-redundant (nr) database, considering as parameter just hits with an identity ≥ 90%. The e-value, query cover and score parameters of every hits were described in supplemental material (S5 Table) [41].

To verify the presence of essential genes among our dataset, the DEG 15.2 database was used [42]. We adopted the BLOSUM62 matrix, score 100 and *e-value* 1e^-05^, using as a query a multifasta file containing the amino acid sequences with the 132 HPs versus the following available genomes belonging to the filo Firmicutes: *Bacillus subtilis* 168, *Bacillus thuringiensis* BMB171, *Staphylococcus aureus* N315 and *Staphylococcus aureus* NCTC 8325. On DEG, each gene has an identification from the single access number assignment, in addition to a reference number, sequence and function.

### Determination of the sub-cellular localization

We used PSORTdb 3.0 [43] and CELLO 2.5 [44,45] to assign the location of the HPs in the cell. PSORTdb is a database that contains information through the association of both laboratory experimentations and computational prediction [46]. CELLO uses a two-level support vector machine (SVM), which involves a number of SVM classifiers. The second one uses a jury SVM to evaluate the outputs from the first level and generate the final probable sub-cellular localization [45]. The prediction of transmembrane helices and protein topology was performed through TMHMM 2.0 [47] and HMMTOP 2.0 [48], and SignalP 4.1 was used to verify the presence of signal peptide cleavage sites [49].

### Prediction of physicochemical parameters

Molecular mass, theoretical isoelectric point (pI), amino acid composition, atomic composition, extinction coefficient, instability index, aliphatic index and high average hydrotherapy were predicted using the ProtParam tool [50], which allows for the calculation of several physicochemical parameters of the proteins.

### Protein-protein interaction network building

For the prediction of PPIs the STRING 10.5 database [51] was used, in which the amino acid sequences of *E*. *antarcticum* B7 were submitted to identify, by similarity, the described PPIs. This database includes direct (physical) and indirect (functional) associations through a computational forecast. To guarantee the reliability of the PPIs, we selected only the most reliable and experimental interactions with score values above 0.700.

Subsequently, the identified interactions were transferred to *E*. *antarcticum* B7 by the interolog mapping method, described in previous studies such as Yu et al. (2004) [44] and Folador et al. (2016) [52]. This method is based on the assumption that if two proteins interact, the orthologous pairs also interact [53,54]. To identify the homology between the proteins, the BLAST against STRING proteins was performed [55], in which we considered only reciprocal hits with a conserved interaction score (IS) greater than 0.5625, corresponding to a 75% identity and 75% coverage, as described by Folador et al. (2016) [52]. The IS value is calculated by multiplying the identity and coverage of the BLAST alignment. Thus, for an interaction, four alignments are performed (two reciprocal alignments among STRING and *E*. *antarcticum*), with IS being the smallest value of these alignments: IS = lowest (lowest (lower (A,a), lower (a, A)), lowest (lower (B, b) lower (b, B)), respectively, representing the "A" and "B" the proteins of STRING and the "a" and "b" proteins of *E*. *antarcticum* B7.

The validation of PPI networks was performed in the Cytoscape 3.6.1 program [56] with the Network Analyzer plugin [57]. This program is a large-scale general-purpose modeling platform for network integration, allowing the visualization and analysis of PPI networks, where protein molecules and molecular interactions are assigned to nodes and edges, respectively. The Network Analyzer computes several topological parameters from networks, such as the node degree distribution and the shortest path, both of which were used in this work.

### Determination and validation of three-dimensional structures

Three-dimensional homology modeling of the target proteins was performed by the MODELLER 9.13 program [58]. To construct the target structures obtained from PDB, we considered only templates with an identity ≥ 30% through BLAST alignment. The models were evaluated according to their stereochemical qualities by PROCHECK 3.5.4 [59], using a range of resolutions from 1.96 Å to 2.70 Å according to the template selected for each protein. The output files in .pdb format were visualized in the UCSF Chimera 1.1.2 [60].

### Performance assessment

To verify the accuracy of the predicted functions for the HPs from the *E*. *antarcticum* B7 genome, a receiver operating characteristic (ROC) was performed [61]. ROC has been extensively used to the analyze the accuracy of the prediction [21]. We randomly selected 100 proteins with known functions and gene names from *E*. *antarcticum* (S2 Table) to be carried by the ROC. These proteins were annotated using the same pipeline described above for the prediction of HPs. The diagnostic efficacy was evaluated on six levels. To classify the prediction, the binary numerals “1” and “0” were used, where “1” denotes a true positive and “0” denotes a true negative. For confidence rating, the integers “2”, “3”, “4” and “5” were used [21,62]. The classification data were submitted to online software ROC Analysis: Web-based Calculator for ROC Curves, which calculates the accuracy, sensitivity, specificity and the ROC area of the functional prediction of the HPs [63]. The average accuracy obtained by the used pipeline was 95.7% (S3 Table). The results from the ROC analysis indicated the high reliability of the set of bioinformatics tools used in our study.

### Arsenic tolerance assay

Since proteins involved in arsenic (As) tolerance were predicted among the HPs analyzed in this study, the strain tolerance to this compound was evaluated. The arsenic stock solutions were prepared in distilled water and sterilized. TSA plates (Tryptic Soy Agar, Merck, Germany) supplemented with arsenic in different concentrations (As; 50, 100, 300, 600, 1000, 1200, 1500 and 2500 μg/ml As Na_2_AsO_4_) were spot-inoculated (3 spots of 10 μL) with 10^4^, 10^5^ and 10^6^ cells mL^-1^ (prepared from an exponential growth phase culture). Triplicate plates were prepared for each metal concentration. The cultures were incubated at 25°C. Each plate was checked for growth after five days, and positives were recorded by the appearance of colonies on at least one spot at the plate surface. The lowest concentration that prevented growth was considered the minimal inhibitory concentration (MIC). The reference strain *Escherichia coli* ATCC 25922 was included for quality control.

## Results and discussion

### Analysis of the hypothetical proteins from the *E*. *antarcticum* B7 genome

The first complete genome to be sequenced for the species, *E*. *antarcticum* B7, was deposited in the NCBI database in 2012 by Carneiro and colleagues. On that date, a total of 2.772 protein-coding genes were predicted on this genome. Of these, 765 (27.59%) were termed hypothetical proteins. In a previous study to investigate the gene expression of *E*. *antarcticum* B7 during cold adaptation, 66 of these HPs were functionally annotated [13]. This indicates that nearly one-quarter of the proteins encoded by this genome still need to be functionally characterized. Therefore, in this work, we have assigned functional information to HPs by the association of several bioinformatics resources.

For this analysis, we first performed a preliminary prediction using the GO FEAT platform. After GO FEAT analysis, 304 HPs of known protein domain and/or families, and their GO terms were selected. Domains are structural, functional and evolutionary units of a protein that are usually responsible for a particular function of a protein; therefore, the knowledge of a protein domain is helpful in understanding its role within a cellular context. This pool of 304 proteins was extensively analyzed using CDART, SMART, Pfam, SUPERFAMILY, SVMProt, and InterPro. The results obtained from the prediction tools are presented in the S4 Table and were analyzed aiming to assign functions to HPs, as described. Functional annotation was assigned with strong confidence to the proteins that exhibited similar function predictions from three or more programs. Thus, we inferred the function of 132 HPs with high confidence (Table 1), where 36 have homologous sequences in the NCBI database with no product function described (S5 Table). Domain identification is important to determine the function of a protein because it is a distinct, functional, and stable structural unit of the protein that is highly conserved during the evolution process [64].

**Table 1:** Hypothetical proteins functionally annotated from *E*. *antarcticum* B7.

No	HP ID	InterPro ID	Protein function
1	Eab7_0026	IPR010375	Cyclic-di-AMP receptor
2	Eab7_0139	IPR014046	Diadenylate cyclase
3	Eab7_0143	IPR028951	Immunity protein 64
4	Eab7_0150	IPR016898	Polyphosphate kinase 2
5	Eab7_0152	IPR006750	Inner membrane exporter, YdcZ
6	Eab7_0154	IPR006750	Inner membrane exporter, YdcZ
7	Eab7_0255	IPR000644	Cystathionine-beta synthase associated with the CorC_HlyC domain
8	Eab7_0278	IPR027417	P-loop containing Nucleoside Triphosphate Hydrolases
9	Eab7_0284	IPR005524	ArsP_1 Superfamily
10	Eab7_0378	IPR006750	Inner membrane exporter, YdcZ
11	Eab7_0379	IPR006750	Inner membrane exporter, YdcZ
12	Eab7_0392	IPR007621	TPM_phosphatase
13	Eab7_0417	IPR025889	Heat induced stress protein YflT
14	Eab7_0504	IPR002696	Haemolytic
15	Eab7_0509	IPR000644	Cystathionine-beta synthase associated with the CorC_HlyC domain
16	Eab7_0515	IPR016047	Peptidase family M23
17	Eab7_0609	IPR000620	EamA-like transporter Family
18	Eab7_0622	IPR001509	NAD-dependent epimerase/dehydratase
19	Eab7_0636	IPR029348	Nucleotidyltransferase-like
20	Eab7_0655	IPR010368	Control of competence regulator ComK, YlbF/YmcA
21	Eab7_0707	IPR029063	S-adenosylmethionine-dependent methyltransferases (AdoMet_Mtases)
22	Eab7_0714	IPR007354	Carotene biosynthesis associated membrane protein
23	Eab7_0741	IPR004394	Ribosomal silencing factor RsfS
24	Eab7_0770	IPR029063	Phosphotransferase system, EIIC
25	Eab7_0774	IPR029063	S-adenosylmethionine-dependent methyltransferases (AdoMet_Mtases)
26	Eab7_0783	IPR025945	SGNH hydrolase-like domain, acetyltransferase AlgX
27	Eab7_0789	IPR002810	NfeD-like C-terminal, partner-binding
28	Eab7_0806	IPR006901	tRNA (adenine-N1-)-methyltransferase
29	Eab7_0807	IPR002678	GTP cyclohydrolase 1 type 2/Nif3
30	Eab7_0823	IPR010708	5' nucleotidase, deoxy (Pyrimidine), cytosolic type C protein
31	Eab7_0861	IPR001763	Rhodanese-like domain
32	Eab7_0909	IPR009474	Disulphide isomerase
33	Eab7_0918	IPR010343	Aromatic acid exporter family member 1
34	Eab7_0931	IPR000620	EamA-like transporter Family
35	Eab7_1015	IPR010368	Control of competence regulator ComK, YlbF/YmcA
36	Eab7_1031	IPR010343	Aromatic acid exporter family member 1
37	Eab7_1052	IPR011010	DNA breaking-rejoining enzymes/ phage integrase
38	Eab7_1085	IPR025889	Heat induced stress protein YflT
39	Eab7_1094	IPR018958	SMI1 / KNR4 family protein
40	Eab7_1169	IPR014719	Ribosomal protein L7/12, C-terminal domain
41	Eab7_1187	IPR007344	GrpB protein
42	Eab7_1213	IPR000620	EamA-like transporter Family
43	Eab7_1218	IPR018958	SMI1 / KNR4 family protein
44	Eab7_1252	IPR004360	Glyoxalase I (lactoylglutathione lyase)
45	Eab7_1290	IPR000160	Diguanylate cyclase (GGDEF domain)
46	Eab7_1307	IPR032713	Multidrug transporter EmrE and related cation transporters [Defense mechanisms]
47	Eab7_1311	IPR000160	Diguanylate cyclase (GGDEF domain)
48	Eab7_1319	IPR004446	D,D-heptose 1,7-bisphosphate phosphatase
49	Eab7_1321	IPR002549	Transmembrane protein TqsA-like
50	Eab7_1322	IPR010162	Zinc peptidase like protein
51	Eab7_1333	IPR001845	HTH ArsR-type DNA-binding domain
52	Eab7_1355	IPR003010	Carbon-nitrogen hydrolase
53	Eab7_1362	IPR012259	Dihydrofolate reductase (DHFR)
54	Eab7_1372	IPR001845	Bacterial regulatory protein/Helix-turn-helix, arsR Family
55	Eab7_1390	IPR005545	YCII-related domain
56	Eab7_1403	IPR009267	Nucleotidyltransferase
57	Eab7_1406	IPR029068	Glyoxalase/Bleomycin resistance protein/Dihydroxybiphenyl dioxygenase
58	Eab7_1409	IPR034660	DinB superfamily protein
59	Eab7_1438	IPR016040	Rossmann-fold NAD(P)(+)-binding proteins
60	Eab7_1462	IPR000330	SNF2 family N-terminal domain
61	Eab7_1478	IPR026881	WYL domain
62	Eab7_1498	IPR005182	Bacterial PH domain
63	Eab7_1499	IPR005182	Bacterial PH domain
64	Eab7_1514	IPR012544	Bacterial PH domain
65	Eab7_1515	IPR005545	YCII-related domain
66	Eab7_1541	IPR005532	Sulfatase-modifying factor enzyme 1
67	Eab7_1554	IPR029072	Transcriptional regulator, YebC-like
68	Eab7_1581	IPR000160	Diguanylate cyclase (GGDEF/EAL domain)
69	Eab7_1590	IPR003744	Vitamin uptake transporter
70	Eab7_1596	IPR000644	Cystathionine-beta synthase associated with the CorC_HlyC domain
71	Eab7_1603	IPR021027	Transposase DNA-binding domain
72	Eab7_1639	IPR017941	Rieske [2Fe-2S] iron-sulfur protein
73	Eab7_1666	IPR006158	Cobalamin (vitamin B12)-binding domain/Transcritional regulator MerR
74	Eab7_1668	IPR004589	ATP-dependent DNA helicase RecQ
75	Eab7_1669	IPR029491	Helix-turn-helix domain
76	Eab7_1675	IPR000524	GntR family transcriptional regulator
77	Eab7_1682	IPR011765	Peptidase M16
78	Eab7_1689	IPR004038	Ribosomal protein L7Ae/L30e/S12e/Gadd45 family
79	Eab7_1718	IPR012826	Flagellar motor switch FliN
80	Eab7_1743	IPR029025	Flagellar biosynthetic protein FlhB
81	Eab7_1766	IPR004007	Dihydroxyacetone kinase family
82	Eab7_1781	IPR008532	Fibronectin-binding protein A N-terminus (FbpA)
83	Eab7_1829	IPR010368	Control of competence regulator ComK, YlbF/YmcA
84	Eab7_1847	IPR003714	Phosphate starvation-inducible protein PhoH, predicted ATPase [Signal transduction mechanisms]
85	Eab7_1870	IPR005119	LysR substrate binding domain
86	Eab7_1875	IPR004155	PBS lyase HEAT-like repeat
87	Eab7_1922	IPR006345	ATP-dependent RecD-like DNA helicase
88	Eab7_1928	IPR021886	MgsA AAA+ ATPase C terminal
89	Eab7_1950	IPR013196	Helix-turn-helix transcriptional regulator/ 3H domain
90	Eab7_1971	IPR001482	Type II secretion system (T2SS), protein E, N-terminal domain
91	Eab7_1974	IPR012902	Prokaryotic N-terminal methylation site
92	Eab7_2007	IPR021722	YvbH-like oligomerisation region
93	Eab7_2029	IPR003838	FtsX-like permease Family
94	Eab7_2032	IPR001537	SpoU rRNA Methylase Family
95	Eab7_2049	IPR005467	Histidine Kinase A
96	Eab7_2065	IPR025618	YtpI-like protein
97	Eab7_2066	IPR000644	Cystathionine-beta synthase
98	Eab7_2073	IPR003356	N-6 DNA Methylase
99	Eab7_2103	IPR025711	PepSY domain
100	Eab7_2119	IPR023753	Oxidoreductase
101	Eab7_2173	IPR025889	Heat induced stress protein YflT
102	Eab7_2195	IPR024775	DinB superfamily protein
103	Eab7_2221	IPR027417	AAA ATPase domain
104	Eab7_2247	IPR002882	2-phospho-L-lactate transferase
105	Eab7_2260	IPR001478	PDZ domain
106	Eab7_2266	IPR003740	YitT_membrane Superfamily protein
107	Eab7_2301	IPR003740	YitT_membrane Superfamily protein
108	Eab7_2309	IPR007809	Flagellar biosynthesis protein FlgN
109	Eab7_2341	IPR003838	FtsX-like permease Family
110	Eab7_2372	IPR000361	Iron-sulphur cluster biosynthesis
111	Eab7_2389	IPR012336	Thioredoxin
112	Eab7_2406	IPR016181	Acetyltransferase
113	Eab7_2419	IPR01176	ATP-grasp family
114	Eab7_2464	IPR024596	DNA-directed RNA polymerase subunit beta
115	Eab7_2498	IPR003838	FtsX-like permease Family
116	Eab7_2499	IPR005467	Two-component sensor histidine kinase
117	Eab7_2500	IPR001789	DNA-binding response regulator
118	Eab7_2518	IPR029046	Lipoprotein localisation LolA/LolB/LppX
119	Eab7_2549	IPR005624	Haem_degrading Superfamily protein
120	Eab7_2581	IPR028974	TSP type-3 repeat
121	Eab7_2599	IPR002563	Flavin reductase like domain
122	Eab7_2611	IPR003442	tRNA A37 threonylcarbamoyladenosine biosynthesis protein TsaE
123	Eab7_2638	IPR019660	Bacterial sensory transduction regulator, YbjN
124	Eab7_2641	IPR022791	Phosphatidylglycerol lysyltransferase
125	Eab7_2678	IPR002781	Sulfite exporter TauE/SafE
126	Eab7_2712	IPR018445	Phosphate transport regulator
127	Eab7_2737	IPR029058	Alpha/beta hydrolase family
128	Eab7_2789	IPR005122	Uracil-DNA glycosylase-like
129	Eab7_2816	IPR011701	Major facilitator superfamily
130	Eab7_2819	IPR007612	LURP-one-related
131	Eab7_2832	IPR018604	Regulatory protein YycI
132	Eab7_2855	IPR009366	Biofilm formation stimulator VEG

In addition, we predicted essential genes using DEG, a database that accommodates *in vivo* and *in vitro* experiments to identify essential genes in eukaryotes and prokaryotes. These genes are fundamental for the cellular machinery acting in the essential processes of the cell [42]. Through the DEG results, it was possible to identify 26 homologous genes, as shown in S6 Table.

**Fig 2:**
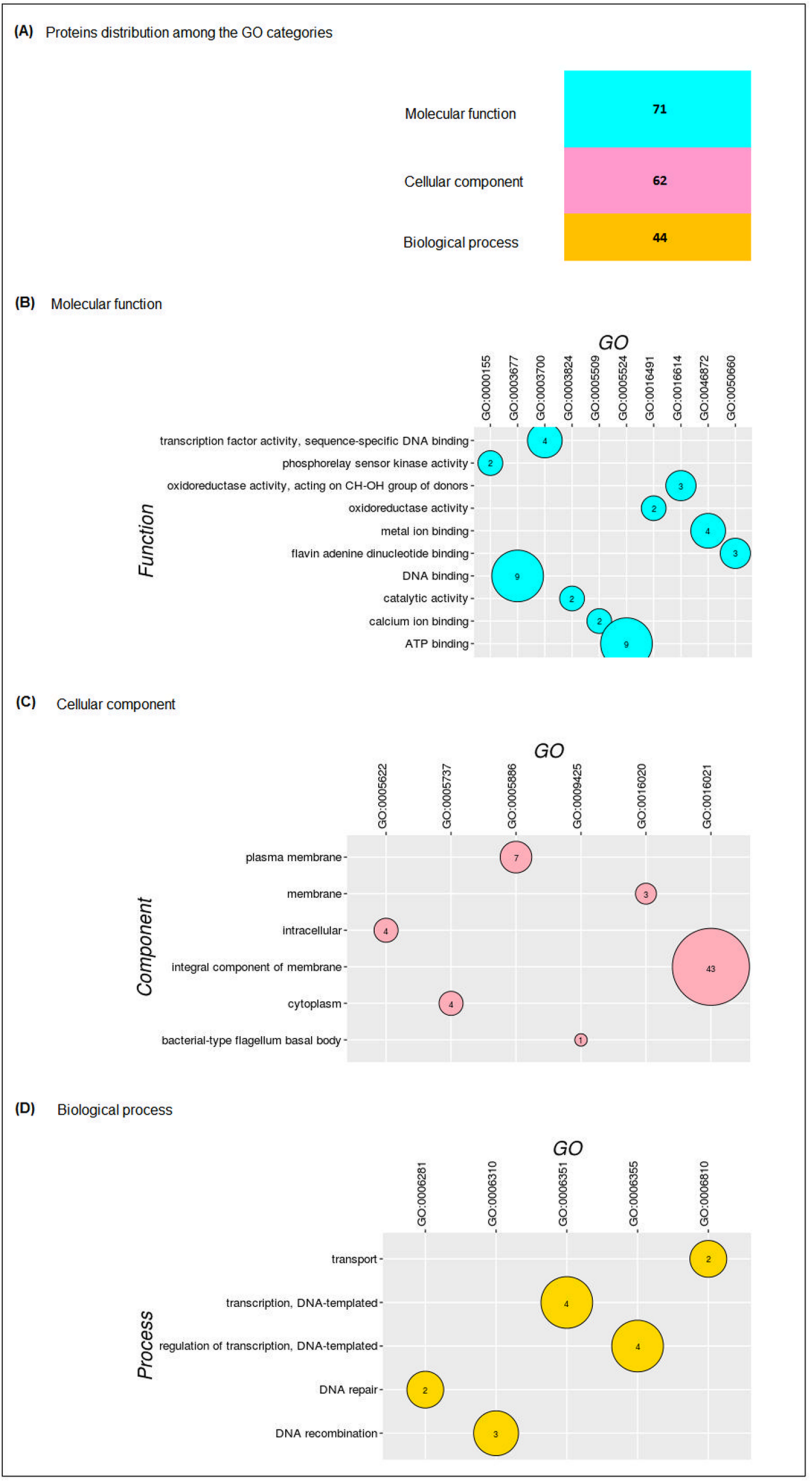
Distribution of the hypothetical proteins of *E*. *antarcticum* B7 among the GO categories. (A) The figure shows how the HPs were distributed among the three gene ontology categories determined by the GoFeat software. (B) Graph of the molecular functions. (C) Graph of the cellular components. (D) Graph of the biological processes.

There were identified GO term predictions for 85 proteins. Fig 2 shows the distribution of the proteins that presented two or more HP in each category, within the three GO categories: biological process, molecular function and cellular component. The GO terms that were represented by only one protein and 47 proteins with no GO terms can be seen in the S7 and S8 Tables, respectively. For molecular function, we identified 71 different GO terminologies designating protein functions; most of these referred to protein binding such as DNA, ATP, and metal binding (Fig 2B and S7 Table). The cellular component category contained 62 different GO terminologies, including 53 that were involved in membrane function and 43 that were integral components of the membrane (Fig 2C). The importance of bacterial membrane proteins in the physiology of Gram-positive bacteria is well-established [65]; however, several membrane proteins are difficult to characterize due to challenges in the preparation of stable membrane proteins with the preservation of their native structure for further studies. The cell membranes act as the front line in the interaction between the cell and the environment [66], so the identification of these membrane proteins, once known as hypothetical, may be the key of understanding the *E*. *antarcticum* B7 mechanisms that enable this psychotropic bacterium to survive under inhospitable temperatures [9].

According to the GO annotation, the biological process category revealed 44 GO terminologies with more representative terms related to transcription, transport, DNA repair, and DNA recognition (Fig 2D). The interactions between the DNA molecule and proteins are in the center of many biological processes [67]. The regulation of transcription represents a vital process for any living organisms, as the control of transcription allows the cell to respond to intra- and extracellular signals, such as environmental stimuli or nutrient scarcity. Among these proteins, Eab7_1675 was functionally annotated as a GntR family transcriptional regulator. This family of transcriptional factors, named gluconate-operon repressors, was first described in *Bacillus subtilis* in 1991 and is a large group of proteins involved in the regulation of several biological processes.

The sequence of Eab7_2372 was predicted as a protein belonging to the iron-sulfur cluster (ISC) biosynthesis process. This cluster of gene functions in eukaryotic and prokaryotic organisms and is required in some biological functions, such as in DNA synthesis for the repair of regulatory processes and redox and non-redox system catalysis [68]. In Gram-negative bacteria, its main role is related to the capture of sulfur and iron atoms for storage and mounting for Fe/S cluster formation; these are used as the final protein receptors [69].

Additionally, Eab7_2641 was annotated as phosphatidylglycerol lysyltransferase (*mprF*), an enzyme known to protect bacteria from cationic antimicrobial peptides that adds L-lysine to phosphatidylglycerol and in this way increases the net positive charge of the bacterial surface and decreases the binding of daptomycin and some cationic antimicrobial peptides, thus contributing to bacterial virulence. This mechanism has been described as a resistance system in *Staphylococcus aureus* [70].

Another protein function identified was the LysR substrate-binding domain (Eab7_1870). LysR regulators are global transcriptional regulators that can act either as activators or repressors of a single gene or an operon [67,71]. The classification of the proteins with no GO terms can be observed in the S8 Table; they are distributed mostly between enzymes, binding proteins and regulatory proteins.

### Prediction of physicochemical properties and sub-cellular localization

In our study, the amino acid sequences of all 132 HPs were analyzed to assess their physico-chemical parameters, and the results can be observed in S9 Table. However, we paid close attention to the proteins that revealed functions related to adaptation and biotechnological interest. The proteins Eab7_0284, Eab7_0655, Eab7_1015, Eab7_1666 and Eab7_2855 all had molecular weight values between 14577.4 and 33132.9. The isoelectric point is the point at which the amino acid of the protein does not tolerate liquid charge, and therefore does not move in an electric field of a direct current. This parameter is used to determine the protein load [22,50,72]. For this group of proteins, it ranged from 4.27 to 10.0. In combination these two parameters help in the visualization of two-dimensional electrophoresis gels (2D), contributing to the laboratorial investigation of these proteins [34].

The aliphatic index is directly related to the molecular fraction of some amino acids and is associated with protein thermostability; that is, the higher its value, the higher the temperature range for which this protein will be stable [22,73,35]. Protein Eab7_2855, which is involved in biofilm formation, had one of the highest aliphatic index values, of 133.14. The grand average of hydropathy (GRAVY) reveals the protein interaction with water, which occurs better with low GRAVYs [22,73]. In Eab7_0655, Eab7_1015 and Eab7_1666, the GRAVY values are between -0.111, -0.490 and -0.678. For the instability investigation, the instability index was applied. This parameter offers an assumption of protein stability in a test tube. To discriminate between stable and unstable proteins, we use as cutoff values >40 and <40, respectively [50,74]. From the proteins of interest, Eab7_0655 (52.91), Eab7_1015 (42.24) and Eab7_1666 (40.20) were considered to be stable. The physicochemical characterization and sub-cellular localization analysis contribute to the elucidation of proteins predicted as proteins of unknown function [19]; this knowledge corroborates the findings of *in vitro* experiments with bacteria that exhibit biotechnological interest, such as *E*. *antarcticum* B7.

The sub-cellular localization of a protein plays an important role in the determination of its function, mainly because protein function is typically correlated to its location [45,62]. Therefore, the knowledge of a protein’s localization in the cellular space is helpful to unveil proteins with unknown function [22,75,76]. The sub-cellular localization can be inferred from the composition in amino acids due to evolutionary adaptation to different sub-cellular sites [75,77]. The proteins Eab7_0284, Eab7_0655, Eab7_1015, Eab7_1666 and Eab7_2588 were predicted to be in the cytoplasm. Proteins in this cellular localization are involved in functional processes such as biosynthesis and transport, which contributes to the secretion of substrates or even other proteins. In cases of environmental bacteria, this process can help in the competition between bacteria inhabiting the same ecological niche [78].

These types of analyses have already been carried out in studies of pathogenic bacteria. In this study 132 HPs were analyzed for their physicochemical parameters and sub-cellular localization. In environmental bacteria, studies with this theme have not yet been reported in the literature, making this study a pioneer.

### Predicted proteins with adaptational functions to extreme environments

Among the 132 proteins functionally annotated in our study, we identified 10 that play an important role for the *E*. *antarcticum* B7 adaptation that allows theses bacteria to survive in extreme environmental conditions.

The protein Eab7_0284 was annotated as the ArsP_1 superfamily, which is a permease encoded by the arsenic resistance operon already identified in *Campylobacter jejuni* [79,80] and that has been recently identified as an organic arsenic transporter [79]. Microorganisms have developed mechanisms to survive in arsenic-contaminated environments, usually lowering arsenic concentrations in the cell by regulating the arsenic uptake, expulsing, or metabolizing arsenic to less toxic compounds [80,81]. Studies have revealed the presence of numerous genes involved in metal and metalloid response, including arsenic resistance, in the genome of species from the *Exiguobacterium* genus [82]. However, *arsP* was not yet identified. The permease ArsP together with ArsB, already described in *E*. *antarcticum* B7 [83], are commonly used to eliminate arsenic toxins in some bacteria through resistance pathways [84]. In general, these pathways act in three ways: reducing the concentrations of arsenic in the cytoplasm that can lead to the metabolism of arsenic into less toxic compounds, limiting its absorption and/or causing its expulsion [81]. A previous study revealed that most of the genes described for other strains of this genus that were considered highly resistant to arsenic, were absent in *E*. *antarcticum* B7 [82]. This indicates that *arsP* might be of great importance to this strain. Two other proteins were functionally predicted for this strain as related to arsenic resistance regulation (Eab7_1333 and Eab7_1372).

The protein Eab7_2599 was predicted to contain a Flavin reductase like domain. Flavin (oxy)reductases stimulate the reduction of flavin by NA(P)H. Among the functions of this protein family is the reduction of cobalamins (III), ferrisiderophores and chromate (III). The latter is a highly toxic compound, with carcinogenicity and mutagenicity properties, that has been described in studies related to bioremediation and which can be harmful in mineral and industrial processes that lead to serious environmental problems and health. *E*. *antarcticum* B7 resistance to chromium confers advantages to its adaptation and may be a future target in bioremediation trials against this toxic metal [85,86].

The protein Eab7_0714 was identified as related to the carotene function biosynthesis associated membrane protein. In non-photosynthetic bacteria this protein protects against ultraviolet (UV) radiation and may act as a regulator of membrane fluidity in these types of environments, interacting with the cell membrane in a similar way to cholesterol. This interaction is dependent on the degree of desaturation and on the length of the lipid chains [87–89].

Through the HPs studied, we identified the proteins that are functionally involved in flagella formation (Eab7_1718, Eab7_1743 e Eab7_2309). The *E*. *antarcticum* B7 flagella operon has already been identified, and its relationship with the adaptation at low temperatures has been inferred. In a study of the gene expression of *E*. *antarcticum* B7, Dall’Agnol et al. (2014) reported the differential expression in genes related to flagella synthesis regulation. They suggested that bacterial motility might be substantial under low temperature conditions.

Proteins involved in biofilm formation were also identified. The proteins Eab7_1015, Eab7_0655 and Eab7_1822 were annotated as the Control of competence regulator ComK and YlbF/YmcA. This family of proteins includes YlbF and YmcA, which are involved in the formation of biofilms and are necessary for correct biofilm formation [90].

The protein Eab7_2855 was annotated as Biofilm formation stimulator VEG. This protein stimulates the formation of the biofilm by inducing the transcription of the *tapA-sipW-tasA* operon, which formats a component of the biofilm, the amyloid fiber (TasA) [91]. Biofilm formation occurs when bacteria switches its state from a free-living form to a surface-associated multicellular state, producing a three-dimensional growth community [91,92]. This structure is supported by a highly hydrated extracellular matrix that is responsible for the adhesion of the cells in the biofilm and to solid surfaces, and serves as a source of nutrient providing carbon, nitrogen, and phosphorus [91]. Biofilm formation of a multicellular bacterial community in a dense barrier is responsible for the high tolerance of these microbial cells to environmental stresses [93,94]. Thus, the biofilm plays a great role for the adaptation of *E*. *antarcticum* in extreme environments.

We found protein Eab7_0741 as Ribosomal silencing factor RsfS. This protein acts by slowing cell growth by inhibiting protein synthesis when the nutrient availability is reduced [95]. RsfS was characterized by *Escherichia coli* and has been demonstrated to slow down or block translation when necessary [96]. The studies conducted in *E*. *coli* propose that RsfS works by binding the large 50S ribosomal subunit [97] so that it prevents 50S and 30S form forming a functional 70S complex [96]. Therefore, it seems to have great importance in the preservation of energy levels during nutritional absences, proving the importance of this protein for the adaptation of *E*. *antarcticum* B7 to the deprivation of nutrient resources in the environment it leaves in.

### Arsenic tolerance in *E*. *antarcticum* B7

Previous research has described the absence in *E*. *antarcticum* B7 of several arsenic resistance genes necessary for arsenic detoxification [82]. However, in our study, we annotated proteins functionally related with arsenic resistance, such as ArsP1 Superfamily (Eab7_0284), together with two other (Eab7_1333 and Eab7_1372) presenting protein domain and family of the transcriptional repressor ArsR, respectively.

Our experimental results indicate that *E*. *antarcticum* B7 is resistant to arsenic, with the ability to grow in all the concentrations tested, up to 2500 μg/mL or 33.5 mM of As. This tolerance level is similar to the ones described for other *Exiguobacterium* strains that possess the genes included in the arsenic resistance operon, reported to grow in arsenic concentrations as high as 10 mM of arsenite (As[III]) and 150 mM of arsenate (As[V]) [82,83,98]. This demonstrates that even though this strain does not contain all of the genes included in the arsenic resistance operon in its genome, it is still highly resistant to arsenic.

Interestingly, we obtained better growth rates in concentrations above 300 μg/mL when compared with the lower concentrations tested. The effect of arsenic in stimulating bacterial growth has been previously reported [99] and has been related to a positive effect on bacteria metabolism, resulting in a shorter generation time and higher cell yield. On the other hand, the observed effect may be related to the addition of sodium (since arsenic was added as Na_2_AsO_4_), which may promote bacterial growth [100].

In nature, bacteria responses to arsenic are different and are usually mediated by genes present in the *ars* operons [101,102]. The most common configuration of this operon includes genes encoding a transcriptional repressor ArsR, an arsenate reductase ArsC, and an arsenite efflux pump [83,103]. Castro-Severyn and colleagues (2017) [82] investigated the presence of the genes responsible for arsenic resistance in 34 genomes within the *Exiguobacterium* genus, using *Exiguobacterium* sp. S17 as a reference due to its confirmed resistance to arsenic [83,98]. According to their study, *E*. *antarcticum* B7 detains only the gene *arsR*, for the arsenical resistance operon repressor, and *arsB*, for the arsenical pump membrane protein, with 50–69% and 85–94% of gene identity compared to the reference, respectively [82]. Thus, the mechanisms that allow *E*. *antarcticum* B7 to tolerate arsenic are still unclear, and proteins such as ArsP, predicted in our study, might be important for this phenotype.

Although arsenic is a natural element, it is a genotoxic component when present at high levels. Indeed, even low concentrations are detrimental to human health. The presence of arsenic detoxifying genes in bacteria is indicative of a potential application in bioremediation processes, for instance, in for the depollution of water effluents [104]. Nevertheless, studies with gene expression and proteomics are still necessary to produce a better understanding of the *E*. *antarcticum* B7 mechanism of arsenic resistance.

### Probable targets with biotechnological interest

We identified some proteins that are functionally involved in processes that can have biotechnological applications, as Eab7_1666 (Cobalamin (vitamin B12)-binding domain), which is related to vitamin B12 synthesis. Vitamin B12 belongs to the cobalamin family and its biosynthesis is restricted to prokaryotes via a complex pathway [105,106]. This vitamin is essential and has been extensively used in the medical and food industries [106]. Its industrial production relies on microbial biosynthetic fermentation, specially using *Pseudomonas denitrificans* and *Propionibacterium shermanii*. Nonetheless, these bacteria present several limitations that make the production of vitamin B12 challenging [105,106].

Researchers have conducted several studies on vitamin B12 engineering. For the industrial production of cobalamin, it is crucial the use of efficient genetic tools and the knowledge of the metabolic pathways in order to improve the production of vitamin B12 [106,107]. Advances in metabolic engineering have been allowed to construct microbial chemical factories; however, the number of microbes that can be used efficiently to produce cobalamin is still small. In this way, the investigation of the *E*. *antarcticum* capability for industrial production of vitamin B12 may be conducted.

Furthermore, given its potential for biotechnology use, studies have suggested that cobalamin also plays an important role in bacterial adaptation to extreme environments, increasing the competitiveness during biofilm formation. However, the protective role of cobalamin has not yet been completely understood [108,109].

The proteins Eab7_0774 and Eab7_0707 were both predicted to be S-adenosylmethionine-dependent methyltransferases, known as AdoMet_Mtases (EC 2.1.1). The Eab7_0806 protein was identified as a tRNA (adenine-N1-)-methyltransferase (EC 2.1.1.217). Methyltransferases are a class of enzymes very present in nature acting in the methylation of biopolymers, as proteins and small metabolites in the three domains of life [110]. These enzymes act by catalyzing a methyl donor group for a receptor molecule, which in turn generates S-adenosylmethionine (SAM-MT) and a modified methylated molecule. The first SAM-MT discovered was catechol, a substance used in the pharmaceutical industry as an anti-cancer and anti-microbial compound [110,111].

Peptidases present biotechnological importance due to their applicability in the industries, acting in the composition of detergents, pharmaceuticals and foods [112]. In this study, proteins comprising the zinc metallopeptidases family were identified. Eab7_0515 belongs to the Peptidase M23 subfamily and has glycylglycine endopeptidase activity. This group also includes some bacterial lipoproteins [113]. Eab7_1322 corresponds to peptidase T, which is a metalloenzyme belonging to the M20 family. This enzyme, under anaerobic conditions, hydrolyzes the tripeptides at their N-termini region. Studies report that the regulation of this gene may contribute to the use of amino acids as energy sources. Håkansson and colleagues demonstrated, that the peptidase T (PepT) is involved in amino acid utilization in *Salmonella typhimurium* [114]. The occurrence of free amino acids is related to the nutritional value, bioactivity, and organoleptic characteristics of the hydrolysates. A protease from *Exiguobacterium* sp. SWJS2 compared with two commercial proteases (papain and alcalase 2.4L), has been reported to have the potential to produce hydrolysates containing peptides or amino acids of nutritional and sensorial importance [115].

The enzyme Eab7_1682 was predicted as an M16 peptidase, present in most prokaryotic and eukaryotic organisms [116]. Frias and colleagues analyzed the membrane vesicles in the extracellular matter, and identified this peptidase was superinduced at 4°C in *Shewanella livingstonensis* NF22^T^, the psychrotolerant bacterium, suggesting that it could be involved in bacterial survival in the Antarctic environment [117].

### Protein-protein interaction network

PPI network analysis was performed for the 11 proteins for cold adaptation and biotechnological interest (Eab7_1743, Eab7_2309, Eab7_0284, Eab7_2599, Eab7_0714, Eab7_1015, Eab7_0655, Eab7_1829, Eab7_2855, Eab7_0741 and Eab7_1666) to evaluate the interactions between them and the other proteins of *E*. *antarcticum* B7. We obtained the network for these proteins individually and in clusters. The interactions within this group of proteins can be observed in the S1 Fig.

The degree of interaction is evaluated according to the color of the nodes on the network; the darker the green, the greater the interaction. The classification in physical and non-physical (regulatory) interactions is represented by the solid and discontinuous lines, respectively. The line thickness represents the IS value, such that thin lines have lower IS and thick lines have greater IS. However, the smaller IS in this network is 0.5625, meaning that all the interactions were mapped with a minimum of 75% identity and 75% coverage. The color of the lines represents the confidence of the interaction, with the yellow/green lines representing a high level of confidence (70% to 90%) and the blue lines representing experimental confidence (>90%) [118].

The protein Eab7_0284, related with arsenic resistance, showed interaction with only 12 proteins, and these interactions were mostly hypothetical. It also presented high confidence interaction with the transcriptional regulator, PadR-like protein (S1 Fig). PadR transcriptional regulators are frequently related with control of detoxification genes [119,120], acting in behaviors such as repressing the phenolic acid decarboxylase gene in *Lactobacillus plantarum* and *Pediococcus pentosaceus* [121,122] and the phenol acid decarboxylase gene in *Bacillus subtilis* [123].

The proteins Eab7_0714 and Eab7_1666 presented interactions with 10 and 12 proteins, respectively (S1 Fig). Eab7_0714 interacted with three phytoene desaturase (Eab7_0708, Eab7_0709 and Eab7_0711) and one phytoene synthase (Eab7_0710), which are proteins involved in the carotenoid biosynthesis pathway [124,125]. Carotenoids are natural organic pigments that are produced by plants, algae, fungi and bacteria in response to various environmental stresses [126]. The interaction revealed between our protein (once known as hypothetical and now assigned as a protein related to carotene biosynthesis) and these proteins reinforces the accurate function attribution performed by this study. The protein Eab7_1666 also showed a high confidence interaction with a protein producer of phytoene synthase (Eab7_0710). Studies have shown that in prokaryotes, such as the extremophilic bacteria from the genera *Deinococcus* and *Thermus*, colalamin, in addition to its role as an enzyme cofactor, is involved in the regulation of gene transcription related to the biosynthesis of carotenoids by binding to CarH repressor [109,127,128]. Carotenoids protect against oxidative damage. Therefore, the presence of cobalamin might provide an advantage in environments that are extremely acidic or highly loaded with metal [109].

The flagella formation proteins Eab7_2309 and Eab7_1743 have been demonstrated to interact with 14 and 61 proteins, respectively (S1 Fig). Both revealed strong interactions with other flagellar synthesis proteins. Eab7_1743 was shown to interact with 16 flagellar proteins that were annotated as part of an operon in the *E*. *antarcticum* B7 genome. Eab7_1743 also exhibited a high-confidence relationship with the gene *sigD* that encoded a sigma factor, which was shown to regulate genes involved in flagella biosynthesis. The expression of the gene *sigD* was induced under cold conditions, suggesting that bacterial motility might be substantial at a low temperature [13].

The proteins related to biofilm formation, Eab7_1829, Eab7_2855, Eab7_0655 and Eab7_1015, interact with 35, 36, 48 and 57 proteins, respectively. Some of these proteins were closely related to the biofilm formation or regulation. For instance, Eab7_1829 exhibited a high degree interaction with the protein encoded by the gene *clpP* that has been described to be involved in biofilm formation. Protein Eab7_0655 showed an interaction with the *ctsR* gene, which is a regulator of the genes *clpC* and *clpP* and is involved in biofilm development in *Staphylococcus aureus* [129]. Eab7_0655 also exhibited a great degree of interaction with *murB*, an up-regulated gene in biofilm formation and maintenance, responsible for the synthesis of cell wall structures [130]. Both Eab7_1829 and Eab7_2855 have demonstrated a high degree of interaction with *coaD*, included in the three-gene operon, and *waaAE-coaD* in *Yersinia pestis*, where *waaA* is a key determinant in biofilm formation [131].

**Fig 3:**
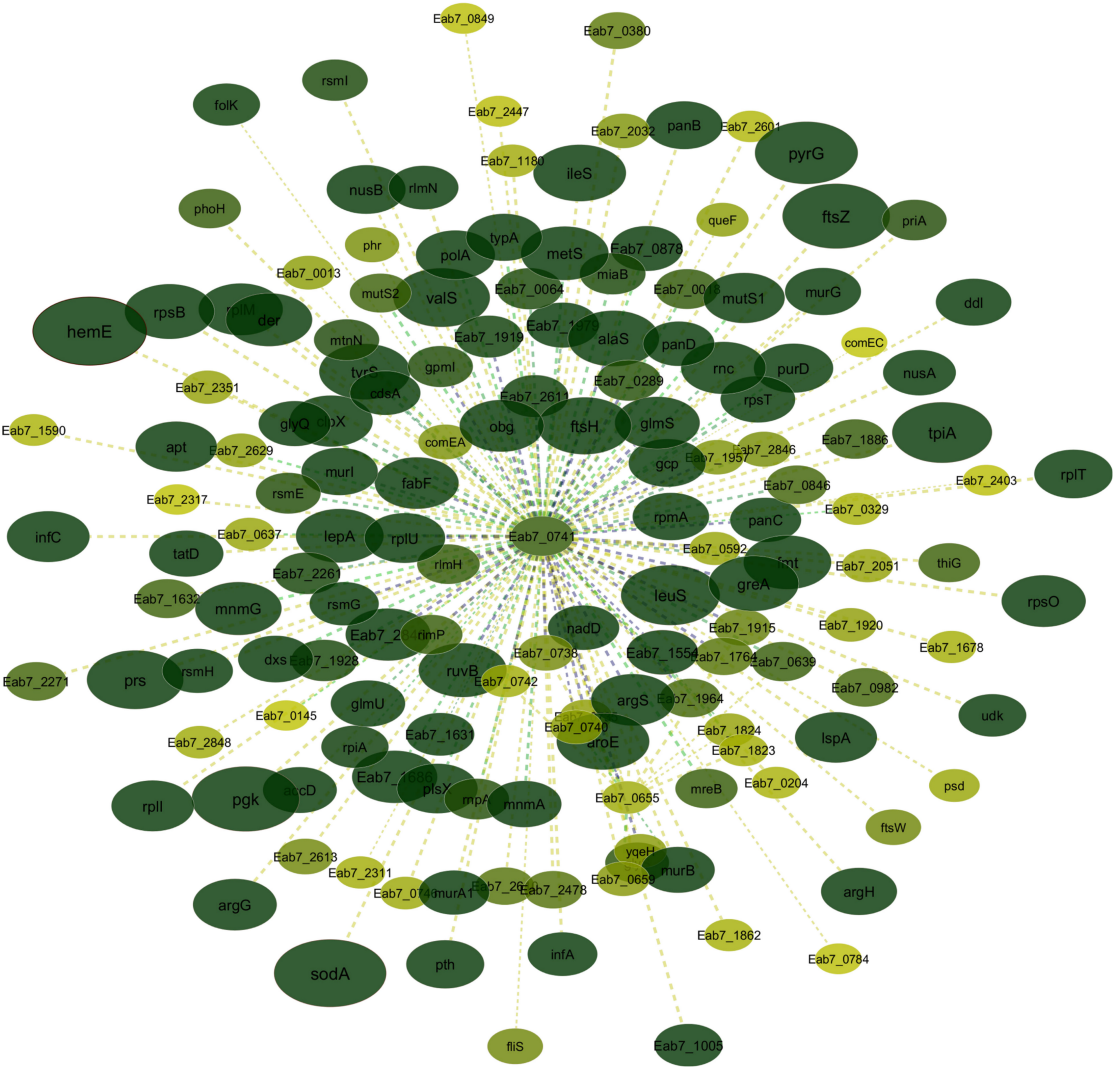
Protein-protein interaction network of protein Eab7_0741, the Ribosomal silencing factor RsfS. The figure shows that the majority of the 156 proteins interacting with Eab7_0741 presents a great number of interactions, which is supported by the node color intensity (dark green), thickness and line color.

The protein with the wider interaction network was Eab7_0741 assigned to the transcriptional regulator RsfS. It is involved in 156 interactions, the great majority of which present a high degree of interaction and confidence. Between these interactions, we identified many proteins related to transcription, such as the 50S and 30S ribosomal proteins, ribosomal RNA large subunit methyltransferases, ribosomal RNA small subunit methyltransferases, tRNA ligases and tRNA synthetase subunits, and elongation factors (Fig 3). Many of the interactions identified support the correct functional prediction of this protein.

Protein cellular functions are determined through their interactions with other proteins and the knowledge of these relationships have led to the development of diverse experimental methods for measurement [132]. Thus, the study of protein-protein interactions are important to infer the function of a completely unidentified protein once its function can be inferred based on the evidence of their interactions with the known proteome of a given organism [64]. Consequently, the identification of the protein interaction is essential since the execution of one function is strongly dependent on the contact or regulatory interaction with another protein [54].

The cellular environment is congested, and the proteins communicate with each other in specific ways leading to cellular processes and biological functions. Therefore, the analysis of PPI networks is required to understand the protein function and the complexity of living systems [54,133]. Functional prediction is one of the main goals of the PPI network, and the availability of PPI networks have helped in the development of computational methods to predict protein functions [64].

### Three-dimensional structures

**Fig 4:**
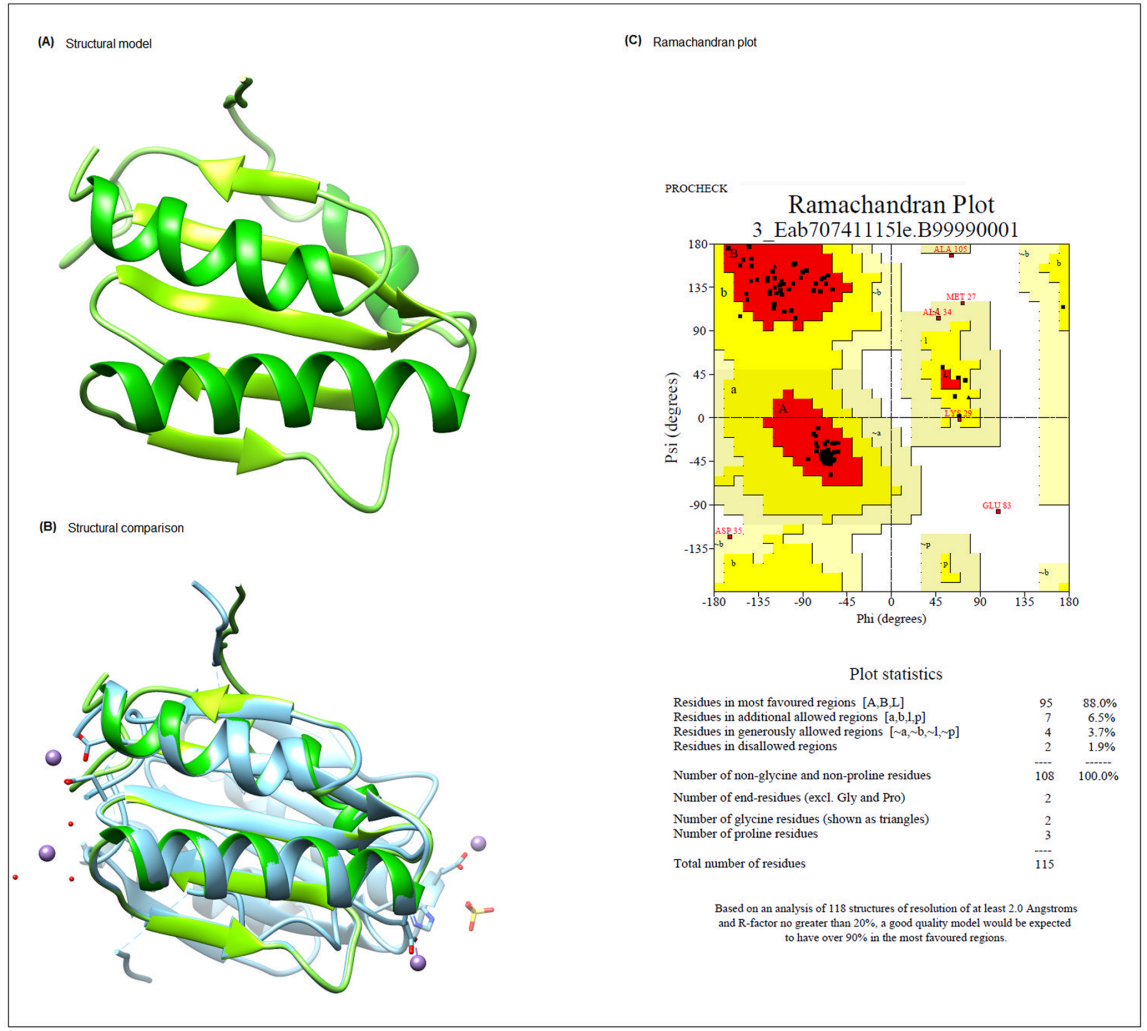
Structural model of protein Eab7_0741, predicted as a Ribosomal silencing factor RsfS. (A) Three-dimensional model obtained from the Protein Data Bank. (B) Alignment between the RsfS protein structures from *B*. *halodurans* and *E*. *antarcticum* generated by MODELLER. (C) Ramachandran plot provided by the PROCHECK program for the RsfS protein from *E*. *antarcticum* B7.

We also constructed the three-dimensional structures for those proteins considered important for the adaptation or potential application in biotechnology. Models for five proteins were obtained (Table 2) with the rate of identity with the model from PDB ranging from 34.53% to 63.46%. The three-dimensional models and the Ramachandran values can be observed in the S2 Fig. Four of the models were constructed from homologous proteins derived from bacteria belonging to the *Bacillus* genus, closely related to the *Exiguobacterium* genus. Based on the value of identity and resolution, the best model obtained was of the protein Eab7_0741, which was annotated as Ribosomal silencing factor RsfS (Fig 4A).

**Table 2:** Three-dimensional structure characteristics for the proteins tested.

HP ID	Protein Name	% Identity	PDB Template	Organism	Method	R-value free	R-value work	Resolution
**Eab7_0741**	Ribosomal silencing factor RsfS	63.46	2o5a	*Bacillus halodurans*	X-ray diffraction	0.278	0.251	2.70 Å
**Eab7_1666**	Cobalamin (vitamin B12)-binding domain	34.53	5c8a	*Thermus thermophilus*	X-ray diffraction	0.227	0.183	2.15 Å
**Eab7_2599**	Flavin reductase like domain	45.17	3bpk	*Bacillus cereus*	X-ray diffraction	0.187	0.153	1.56 Å
**Eab7_1015**	Control of competence regulator ComK, YlbF/YmcA	56.19	2pih	*Bacillus subtilis*	X-ray diffraction	0.254	0.207	2.10 Å
**Eab7_0655**	Control of competence regulator ComK, YlbF/YmcA	54.54	2oee	*Bacillus subtilis*	X-ray diffraction	0.249	0.217	1.96 Å

The model structure obtained for the RsfS protein was previously determined by X-ray crystallography and refined with diffraction data to 2.1 Å resolution from *Mycobacterium tuberculosis*, which was solved by molecular replacement with a truncated poly-Ala model (Ala7-Ala103) derived from an ortholog isolated from *B*. *halodurans* (PDB 2o5a), the same model used in this study to the three-dimensional modeling by homology. RsfS contains the α1-β1-β2-α2-β3-β4-β5-α3 fold and ortholog structures from *Chromobacterium violaceum* (PDB 2id1), *Zymomonas mobilis* (PDB 3ups) and *B*. *halodurans* onto *M*. *tuberculosis* RsfS, demonstrating that the overall structure is well-conserved from the N terminus to the end of β5 [95]. The alignment between the structures from *B*. *halodurans* and *E*. *antarcticum* RsfS generated by MODELLER also shows a high structural similarity (Fig 4B). RsfS is known as component of the protein synthesis regulation system through the binding to the 50S ribosomal protein L14, impairing joining with the 30S ribosomal subunit. Haüser and colleagues reported that the RsfS-interaction epitope of L14 involves the highly conserved residues K114 and T97 as the most important ones, in addition to R98. The residues T97 and R98 are involved in bridge B8, which contacts the 30S ribosomal subunit. The authors performed a docking model to predict the binding of RsfA to these residues, showing that they sterically interfere with ribosome subunit joining, probably blocking translation [96].

To identify the stereochemical characteristics and possible mismatches in the molecule architecture, as well as to confirm the quality of the model, it is important to evaluate the quality of protein structures [134]. The PROCHECK program provided the Ramachandran plot for the Rsfs protein of *E*. *antarcticum* B7, and the results showed that 88% of the residues are in favorable regions, indicating that the generated model presents an excellent degree of reliability (Fig 4C).

The relationship between three-dimensional proteins structures and their biological functions is evident and the knowledge of the protein structure combined with functional annotation methods can lead to the elucidation of uncharacterized proteins [32]. Bioinformatics methods based on available databases have been used to understand protein structure and its function [54]. Therefore, the sequence-to-function method of functional prediction has identified the function of the proteins in this study. The association with the structural information performed for a few groups has confirmed the accurate function annotation.

The model obtained for the RsfS protein predicted from *E*. *antarcticum* B7 was already evaluated experimentally to *M*. *tuberculosis* [95], and our study has shown great results to the model obtained by computational methods of databases comparison of the amino acid sequence of this protein with the ones previously characterized. This kind of comparison is possible because amino acid sequences determine the structure, and the structures commend the biochemical function. In this way, proteins with shared similarity of their amino acid sequence usually perform similar functions [135].

## Conclusions

Proteins are versatile macromolecules that play crucial role in biological processes. The identification of protein functions is fundamental for the understanding of these processes. We used an *in silico* approach to predict the function of hypothetical proteins from the *E*. *antarcticum* B7 genome. We attributed a function to 132 HPs with high confidence. The prediction of sub-cellular localization and physicochemical parameters were useful to reinforce the understanding of the particular characteristics of the proteins annotated. Those proteins were further investigated for their interactions and three-dimensional structures. PPI investigation is important to determine the relationship between these proteins and the known proteome of a given organism, helping to infer correctly its function. We identified the presence of proteins that play important roles in the mechanisms of adaptation to adverse environments, such as biofilm formation, flagellar biosynthesis, transcription regulation, carotenoid biosynthesis, and others. The pipeline used in this study allowed us to obtain excellent results and can be used to assign protein function to hypothetical proteins. We also demonstrated *E*. *antarcticum* B7 resistance to arsenic. Our findings open possibilities for better investigation of this bacterium for application in the biotechnology field.

## Supporting information

S1 Fig Protein-protein interaction of all 11 proteins tested. This image shows how each of the proteins tested interacts with each other and with other proteins form the *E*. *antarcticum* B7.
(TIFF)

S2 Fig Three-dimensional models and Ramachandran values for the proteins Eab7_0655, Eab7_1015, Eab7_2599 and Eab7_1666 from *E*. *antarcticum*. (TIF)

S1 Table List of bioinformatics tools and databases. (XLSX)

S2 Table Dataset of annotation function of 100 proteins functionally known from *E*. *antarcticum* B7 using the same pipeline used for the HP prediction. (XLSX)

S3 Table Accuracy, sensitivity, specificity and ROC area results of tools of the pipeline used for the annotation of 100 proteins functionally known from *E*. *antarcticum* B7 by ROC analysis. (XLSX)

S4 Table Annotation dataset results for the 304 hypothetical proteins from *E*. *antarcticum* B7, submitted to the pipeline with Go feat, Pfam, CATH, SUPERFAMILY, SVMProt, CDART, SMART and InterPro. (XLSX)

S5 Table Results of the Blastp search for similar sequences against non-reduntant (nr) database. (XLSX)

S6 Table Result of essential gene prediction using DEG. (XLSX)

S7 Table List of GO terms represented by only one protein for molecular function and biological process. (XLSX)

S8 Table List of protein with no GO term prediction. (XLSX)

S9 Table List of predicted physicochemical parameters, sub-cellular localization and topology of transmembrane helices for the HPs from *E*. *antarcticum* B7. (XLSX)
