# Supplementary figures and images for: Functional annotation of hypothetical proteins from the Exiguobacterium antarcticum strain B7 reveals proteins involved in adaptation to extreme environments, including high arsenic resistance
Source: PLoS One. 2018 Jun 25;13(6):e0198965. doi: 10.1371/journal.pone.0198965 (PMC6016940; doi:10.1371/journal.pone.0198965)

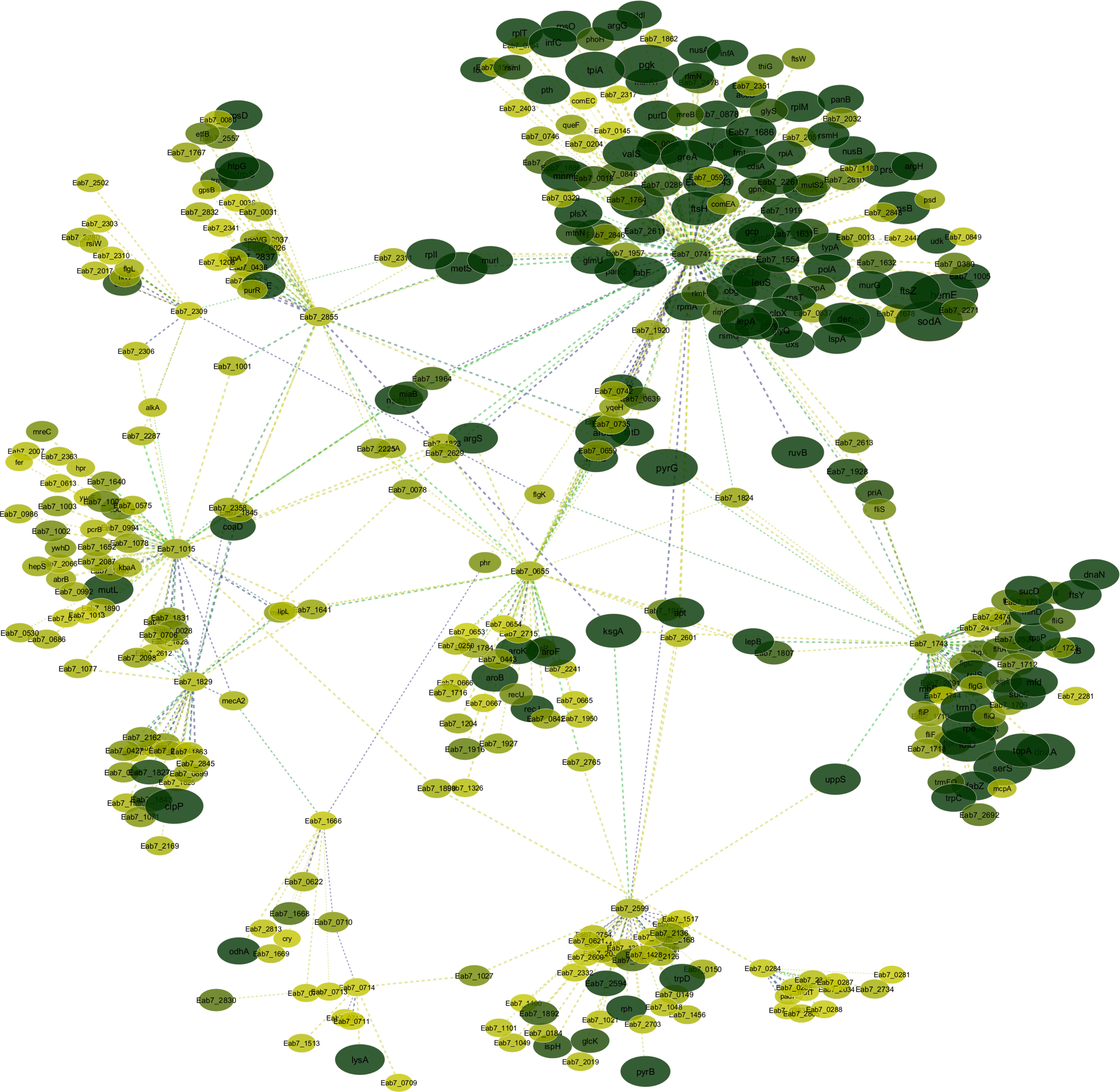

Supplement: S1 Fig — This image shows how each of the proteins tested interacts with each other and with other proteins form the E. antarcticum B7. (TIFF) [file pone.0198965.s001.tiff]
